# Investigating Key Factors Related to the Decision of a Do-Not-Resuscitate Consent

**DOI:** 10.3390/ijerph19010428

**Published:** 2021-12-31

**Authors:** Hui-Mei Lin, Chih-Kuang Liu, Yen-Chun Huang, Chieh-Wen Ho, Mingchih Chen

**Affiliations:** 1 Taipei City Hospital, RenAi Branch Nursing Supervisor, Taipei 106, Taiwan; b0739@tpech.gov.tw; 2 Graduate Institute of Business Administration, Fu Jen Catholic University, New Taipei City 242, Taiwan; 059435@mail.fju.edu.tw (C.-K.L.); hivicky92@gmail.com (Y.-C.H.); cwho1220@gmail.com (C.-W.H.); 3 Artificial Intelligence Development Center, Fu Jen Catholic University, New Taipei City 242, Taiwan; 4 Department of Urology, Fu Jen Catholic University Hospital, New Taipei City 243, Taiwan; 5 Department of Life Science, National Taiwan University, Taipei 106, Taiwan

**Keywords:** do not resuscitate, family palliative care consultation, palliative care, TW-PCST score

## Abstract

Background: The decision to sign a do-not-resuscitate (DNR) consent is critical for patients concerned about their end-of-life medical care. Taiwan’s National Health Insurance Administration (NHIA) introduced a family palliative care consultation fee to encourage family palliative care consultations; since its implementation, identifying which families require such consultations has become more important. In this study, the Taiwanese version of the Palliative Care Screening Tool (TW–PCST) was used to determine each patient’s degree of need for a family palliative care consultation. Objective: This study analyzed factors associated with signing DNR consents. The results may inform family palliative care consultations for families in need, thereby achieving a higher DNR consent rate and promoting the effective use of medical resources, including time, labor, and funding. Method: In this retrospective study, logistic regression analysis was conducted to determine which factors affected the DNR decisions of 2144 deceased patients (aged ≥ 20 years), whose records were collected from the Taipei City Hospital health information system from 1 January to 31 December 2018. Results: Among the 1730 patients with a DNR consent, 1298 (75.03%) received family palliative care consultations. The correlation between DNR consent and family palliative care consultations was statistically significant (*p* < 0.001). Through logistic regression analysis, we determined that participation in family palliative care consultation, TW–PCST score, type of ward, and length of stay were significant variables associated with DNR consent. Conclusions: This study determined that TW–PCST scores can be used as a measurement standard for the early identification of patients requiring family palliative care consultations. Family palliative care consultations provide opportunities for patients’ family members to participate in discussions about end-of-life care and DNR consent and provide patients and their families with accurate medical information regarding the end-of-life care decision-making process. The present results can serve as a reference to increase the proportion of patients willing to sign DNR consents and reduce the provision of ineffective life-prolonging medical treatment.

## 1. Introduction

The main purpose of do-not-resuscitate (DNR) is to ensure patients’ medical autonomy is respected and to prevent ineffective medical treatment [1,2]. DNR policies were first enacted in the United States in 1976. On 7 June 2000, Taiwan’s Ministry of Health and Welfare enacted the *Natural Death Act*, which contains provisions enshrining a patient’s right to choose a natural death [3,4,5].

A persistent problem with consent is that DNR discussions do not occur frequently enough and decisions are often made in the final stage of a patient’s disease, at which point the patient is often unable to make the DNR decision autonomously or lacks effective communication in the hospital (Yuen et al., 2011, [5,6,7,8,9]. Furthermore, cultural background may influence DNR decision-making.

Confucianism and filial piety emphasize family-centric values and close relationships among family members. Therefore, in Confucian-influenced Asian cultures, major illnesses often affect the entire family, not only the patient [10]. To mitigate patients’ psychological distress and physical exhaustion, family members will often actively participate in discussions with physicians, especially when facing a major decision. In situations where patients may not be able to make their own decisions and participate in treatment decision-making, family members have primary authority [10,11,12,13]. Insufficient information can make the decision to adopt a DNR order difficult for patients and their families [2,9,14,15,16,17]. In Western cultures, which tend to be more oriented toward the individual, a patient’s family members often do not directly interfere with the patient’s decisions; however, patients and their families in Western cultures are more willing to make health care decisions in advance, which improves the patient’s quality of life [13,18]. 

Taiwan is the first Asian country to enact palliative care legislation. To improve public awareness of palliative care, Taiwan’s Mackay Memorial Hospital introduced the first hospice care in 1990 [19]. Taiwan’s *Palliative Care Act*, a natural death act enacted in 2000, granted patients the right to refuse cardiopulmonary resuscitation by signing documents known as DNR consents. In 2012, the Taiwan National Health Insurance Administration (NHIA) introduced a family palliative care consultation fee to encourage physicians and medical teams to communicate with the families of terminally ill patients regarding DNR consent and palliative care. In these family consultations, the medical team, the patient, and family members discuss DNR and palliative or hospice care plans that are best suited to the patient. In recent years, palliative care services have steadily increased, and studies have suggested that physicians must discuss palliative care earlier to provide end-of-life patients with a better quality of death [20].

To assess the severity of a case when a patient is admitted, Taipei City Hospital uses the Palliative Care Screening Tool–Taiwan version (TW–PCST) score. Within 24 h of a patient’s admission, nursing staff use the patient’s TW–PCST score to identify whether a family palliative care consultation is required and whether the patient must sign a DNR consent soon after admission to avoid initiation of the decision-making process when the patient is already unconscious or bedridden due to illness. The TW–PCST evaluates four categories: (1) the severity of the baseline disease process, (2) the severity of the comorbidity process, (3) functional performance status, and (4) whether the patient has had frequent admissions or intensive care unit (ICU) stays [21] (Table S1). TW–PCST scores can be used to identify inpatients in need of a family palliative care consultation quickly and to arrange consultations for them in advance.

This study adopted quantitative analysis using large-scale individual data. The hospital information system (HIS) data contained 2144 deceased patient’s clinical records from January to December 2018, and logistic regression was used to analyze which factors affected patients’ DNR decisions. The relationships discovered between these factors and a patient’s palliative care and DNR status. In 2012, Taiwan’s NHIA introduced a family palliative care consultation fee. The results of the present study further support the implementation of the NHIA’s family palliative care policy. Family palliative care consultations that integrate the significant variables identified herein may result in a higher DNR consent rate; thus, this study can serve as a reference for medical professionals to improve patients’ quality of death and promote the signing of DNR consents, thereby improving the efficient use of medical resources.

## 2. Materials and Methods

### 2.1. Study Design and Population

**Figure 1:**
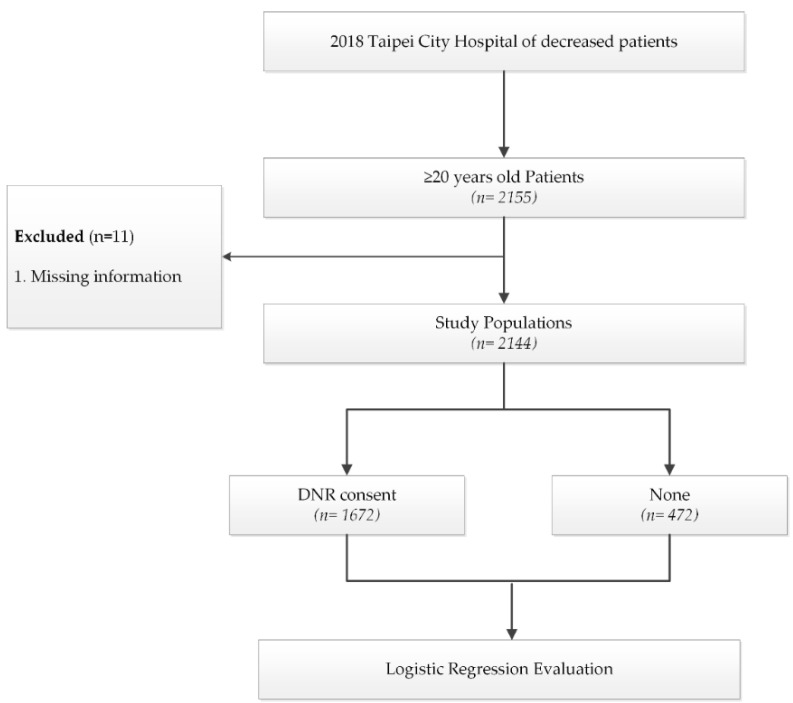
Patient enrollment and exclusion.

In this study, we prioritized deceased patients ≥20 years old selected from the HIS data of Taipei City Hospital for the family palliative care consultations. The data was collected between 1 January and 31 December 2018 (Figure 1). When each patient was admitted, a nursing assessment integrating the TW-PCST and used TW-PCST scores was administered to divide the patients into two groups: those with scores <4 and those with scores ≥4. Those with scores ≥4 were further evaluated by the physician, and the patients and their families were invited to participate in palliative care consultations.

### 2.2. Baseline Variables

The HIS data provided the patients’ detailed medical information, including sex, age, type of ward, family palliative care consultation, TW–PCST scores and length of stay (LOS). The following paragraph explains the variable definitions. (1) Age was stratified into three groups: <65, 65–79, and ≥80. Age 65 is the general cutoff age of older adults, and the ≥80 group was added for a more balanced grouping. (2) Four types of wards were included: respiratory care (RCW), intensive care (ICU), palliative care, and general. Patients who had been transferred from intensive care to the RCW were dependent on respirators, and the length of their hospital stays was more than 21 days. Because their stays were different from those of the other patients, the RCW was included separately from the other wards. (3) LOS: an average length of hospital stay was used. (4) The TW–PCST is a screening tool adapted from St. Mary’s Medical Center in San Francisco, CA, USA. 

### 2.3. Statistical Analysis

The demographic data of the patients were analyzed as categorical variables using a chi-square test, of which the results were expressed as frequencies and percentages. Continuous variables were analyzed using a Student’s *t* test, of which the results were expressed as means and standard deviations. R statistical software was used to perform the statistical analyses. All statistical analyses were two-sided, and *p* < 0.05 was considered statistically significant.

## 3. Results

### 3.1. Study Population Characteristics

A total of 2144 patients were included in this study, as indicated in Table 1. Of the deceased patients, 1240 (57.84%) were men. Regarding the patients’ ages, 1281 (59.79%) were ≥80 years. Regarding the type of ward in which patients were treated, 1142 cases (53.26%) were selected from the general ward, and 667 (31.11%) were selected from the ICU. In addition, 1551 patients (72.34%) participated in family palliative care consultations; 886 (41.32%) had a TW–PCST score of 0–3 points, and 880 (37.31%) had a score of ≥4 points. A total of 458 missing TW–PCST scores were noted in the HIS system; this may be attributed to the heavy work burden of medical staff and the consequent neglect to log data after evaluations. In total, 1009 patients (47.06%) had an LOS of 1–10 days.

### 3.2. DNR Signature Situation

The DNR status results are listed in Table 2. Among the patients who signed DNR consents, 57.17% were men and 42.83% were women. The average TW–PCST score (*p* ≤ 0.001 ***), average LOS (22.99 ± 52.53 vs. 41.36 ± 124.90, *p* = 0.0035 **), and percentage of patients who received family palliative care consultations (75.03 vs. 61.11%, *p* ≤ 0.001 ***) differed significantly between those who had signed and those who had not signed DNR consents.

### 3.3. Factors Affecting DNR Status

According to the results of the logistic regression analysis, seven factors were identified that may affect a patient’s likelihood of signing a DNR consent. As indicated in Table 3, the type of ward in which a patient is treated (*p* ≤ 0.001 ***), family palliative care consultation (*p* ≤ 0.001 ***), TW–PCST scores (*p* = 0.0086 ****), and LOS (*p* = 0.0010 **) are significantly correlated, which suggests that these factors—especially family palliative care consultation and TW–PCST scores—may affect a patient’s DNR status. The coefficient of length of stay was −0.0023, which indicates that a longer LOS was associated with a slightly but significantly lower probability of signing a DNR consent.

## 4. Discussion

In this study, among the 1730 patients with a DNR consent, 1298 (75.03%) had participated in a family palliative care consultation. In the logistic regression analysis, TW–PCST score, type of ward, LOS, and family palliative care consultation exhibited statistically significant correlations with DNR status. Family palliative care consultation can allow dying patients and their families to make end-of-life care decisions together and allow providers to understand a patient’s needs before discussing DNR consent and related health care considerations. Professionals suggest that physicians discuss palliative care with patients and their families at the earliest appropriate time to convey empathy and to ease the process of signing a DNR consent [1,22]. In a study of health care providers’ attitudes toward DNR consent, 57% of the respondents indicated that they believed that providing enough information regarding DNR consent to patients was important. However, the execution rate was only 21% [14,23].

The results of the present study indicate that TW–PCST scores is one of the key decision-making factors that affect patient’s DNR consent. DNR discussions among health care providers, patients and their families do not occur frequently enough [2,24] and often occur later than is appropriate; accordingly, health care providers should prioritize early discussion of a patient’s wishes regarding DNR and related considerations [9,24]. Moreover, older adult patients with chronic diseases often exhibit poor responses to CPR, which limit their ability to participate in DNR decision-making [25,26,27,28]. Studies have demonstrated that various factors affect DNR decision-making, including economic status, suggestions from nurses or physicians, education level, place of residence of family members, and sex [9,14,15]. A tool for evaluating the extent of the reversibility of a patient’s illness that can be used before the patient is admitted to the hospital [29] would be helpful in preparing for DNR discussions. Taipei City Hospital uses TW–PCST scores as a palliative performance scale to record preliminary measurements when a patient is admitted to the hospital. Upon hospital admission, if a patient’s TW–PCST score is ≥4 points, they are prioritized for family palliative care consultation. This policy is helpful for encouraging the early discussion of DNR consent with end-of-life patients and their families. The results of this study can serve as a reference for promoting end-of-life palliative care, and the TW–PCST scores of admitted patients can be used to determine their need for a DNR order or palliative care consultation at an early juncture in hospitalization.

A patient’s likelihood of signing a DNR consent is significantly correlated with having received a family palliative care consultation (*p* < 0.001). In a previous study, 50% of the participating patients expressed their concern that signing a DNR consent would cause them to receive inferior medical care [9,13,30]. DNR decisions become inevitable in many end-of-life scenarios [15]; thus, the provision of correct and balanced medical information to patients and their families is a crucial concern in enhancing the quality of intensive care [26]. In 2019, Pettersson et al. argued that hospitals must provide an appropriate environment for palliative care discussions, enable patients and their relatives to obtain more DNR information from the medical team, and guide all parties toward the optimal decision for the patient [14,23]. Studies have suggested that the failure of DNR policy may be attributed to physicians lacking communication skills and sufficient information to discuss DNR consent with patients. Physicians must provide different perspectives on DNR, discuss DNR consent openly, and consider the opinions and preferences of patients’ family members to increase the effectiveness of their communication and to understand the needs of the patients and their families [23]. Family members play a key role in the decision-making process [9]. Physicians’ failure to provide adequate information about DNR consent to patients or their families, as well as the inability of patients and their families to participate in end-of-life care decisions, might lead to situations in which a patient experiences prolonged suffering before death [2,5,30]. Family relations are close in Asian countries, and toward the end of a patient’s life, the thoughts and feelings of the patient’s family members are a critical concern. Over 70% of patients are willing to learn detailed information about DNR consent and share the DNR decision with their family members [9,31]. In addition, effective, high-quality communication among family members and health care providers can reduce decision-making conflicts [17,32].

In 2012, the Taiwanese NHIA implemented a family palliative care consultation fee and established a platform for medical teams to actively communicate with family members. These measures aimed to foster understanding of the concepts of peaceful end-of-life care and DNR among patients and their families, to provide opportunities for patients’ family members to participate in discussions regarding end-of-life care and DNR, and to provide patients and their families with accurate medical information regarding the end-of-life care decision-making process. Although some medical institutions have enacted DNR policies and some jurisdictions have laws governing the same, studies have indicated that patients and their families often do not understand the content of such policies. Therefore, the DNR decision-making process should be addressed in national policies and laws [33]. Yuen et al. argued that hospitals must amend their cultures and policies and provide communication skills lessons and financial incentives to encourage physicians to participate in more active discussions with patients and their families regarding DNR consent [2]. The support of physicians is of major importance in addressing concerns regarding patients’ and family members’ emotional reactions [15]. Medical teams can publicly discuss their views on DNR decision-making to further inform patients’ family members about the implications of DNR consent and to encourage them to reach consensus on end-of-life care decisions [5,22,23]. Taiwan has incorporated family palliative care consultation into its health care policy, which encourages physicians and other medical staff to provide sufficient information and suggestions to reduce information asymmetry between patients and their families.

## 5. Conclusions

This study conducted a logistic regression analysis and identified four significant variables that affect patients’ DNR consent. The use of the TW–PCST as a measurement tool can help health care providers identify patients in need of DNR consultations early. Family palliative care consultations provide opportunities for patients’ family members to participate in discussions regarding end-of-life care and DNR and to obtain accurate medical information regarding the end-of-life care decision-making process. These results provide a reference and measurement scale for other countries to increase the proportion of patients willing to sign DNR consent and reduced life-prolonging of ineffective medical treatment.

## Figures and Tables

**Table 1:** Demographics of deceased patients at Taipei City Hospital from 1 January 2018, to 31 December 2018.

Baseline		N%	(%)
Sex	Male	1240	57.84
	Female	904	42.16
Age group	<65	285	13.29
	65–79	578	26.96
	≥80	1281	59.79
Type of ward	General	1142	53.26
	Respiratory care	18	0.84
	Intensive care	667	31.11
	Palliative	317	14.79
Diagnosis	Cancer	466	21.74
	Non-cancer	1678	78.26
Family palliative care consultation	Yes	1551	72.34
	No	593	27.66
TW–PCST scores	0–3	886	41.32
	≥4	880	37.31
	Unknow TW–PCST	458	21.36
LOS (days)	1–10	1009	47.06
	11–20	453	21.13
	21–30	276	12.87
	>30	406	18.94

TW–PCST scores: Palliative Care Screening Tool–Taiwanese version scores; LOS: length of stay.

**Table 2:** DNR status from 1 January to 31 December 2018, in Taipei City Hospital HIS (N = 2144).

Baseline		DNR Consent		None		*p*
		(*n* = 1730)		(*n* = 414)		
		*n*	%	*n*	%	
Sex	Male	989	57.17	251	60.63	0.2003
	Female	741	42.83	163	39.37	
Age group	<65	227	13.12	58	14.01	0.8886
	65–79	468	27.05	110	26.57	
	≥80	1035	59.83	246	59.42	
Age mean (std)		80.37 (13.41)		80.25 (13.86)		0.8643
Type of ward	General	871	52.09	271	57.42	0.1630
	Respiratory care	12	0.72	6	1.270	
	Intensive care	530	31.7	137	29.03	
	Palliative	317	100	-	-	
Diagnosis	Cancer	381	22.02	85	20.53	0.5086
	Non-cancer	1349	77.98	239	79.47	
Family palliative care consultation	Yes	1298	75.03	253	61.11	<0.001
	No	432	24.97	161	38.89	
TW–PCST scores	0–3	682	39.42	204	49.28	<0.001
	4+	645	37.28	155	37.44	
	Unknown TW–PCST	403	23.29	55	13.29	
Length of Stay mean (std)		22.99 (52.53)		41.36 (124.90)		0.0035

TW–PCST scores: Palliative Care Screening Tool-Taiwanese version; LOS: length of stay; SD: standard deviation.

**Table 3:** Significance of variables in the logistic regression for DNR status.

Baseline	Estimate	Error	Pr (>Chi)
Intercept	0.7275	0.4060	
Sex	−0.1979	0.1284	0.1900
Age	0.0019	0.0046	0.6625
Type of ward	0.4100	0.0577	<0.001 ***
Family palliative care consultation	0.8236	0.1331	<0.001 ***
Diagnosis	0.1665	0.1562	0.2820
TW–PCST scores	−0.2253	0.0869	0.0086 **
LOS (days)	−0.0023	0.0007	0.0010 **

**: *p* < 0.01; ***: *p* < 0.001; TW–PCST scores: Palliative Care Screening Tool-Taiwanese version; LOS: length of stay.

## Data Availability

The data presented in this study are not available on request from the corresponding author. Due to the General Data Protection Regulation, the data presented in this research are not publicly available.

## Supplementary Materials

The following are available online at https://www.mdpi.com/article/10.3390/ijerph19010428/s1, Table S1: title The calculation of Taiwanese version-Palliative Care Screening Tool (TW-PCST).
