# Supplementary material for: Investigating Key Factors Related to the Decision of a Do-Not-Resuscitate Consent
Source: Int J Environ Res Public Health. 2021 Dec 31;19(1):428. doi: 10.3390/ijerph19010428 (PMC8744657; doi:10.3390/ijerph19010428)
Supplement: Supplementary file 1 [file ijerph-19-00428-s001.zip › ijerph-1469674-supplementary.pdf]

**Table S1. The calculation of Taiwanese version-Palliative Care Screening Tool (TW-PCST)**

A one-page screening tool adapted from the instrument used in St. Mary's medical center in the United States, was initiated on April 2015 among patients admitted to the Taipei City Hospital at the first day of admission. This checklist consists four categories, namely (A) severity of basic disease process, (B) progression of co-morbidity, (C) functional status score according to Eastern Cooperative Oncology Group (ECOG) Performance Status, and (D) frequent exacerbations. In category A, any of the seven selected most prevalent conditions was scored 2 points. These include cancer (metastatic/recurrent), advanced chronic obstructive pulmonary disease (COPD), end-stage liver disease, kidney dialysis (age ≥65 and dialysis for > 2 years), advanced cardiac disease, neurologic disease with severely reduced function (i.e. stroke, coma, dementia resulting in bed-bound), and life-limiting acute illness (i.e. adult respiratory distress syndrome, sepsis, multiple organ dysfunction syndrome). In category B, primary cancer and five varieties of moderate co-morbidities were listed and scored 1 point for each. In category C, ECOG scores vary between 0 and 3 (1=ambulatory and capable of all self-care but unable to carry out any work activities, 2=capable of only limited self-care; confined to bed or chair, 3=completely disabled; cannot carry on any self-care). In category D, frequent exacerbations and use of resources were included, such as frequent hospital admissions and ICU stay, and each was scored 1 point. The total-ABCD score represent the summation of the TW-PCST score, which ranges from 0 to 31.

| Screening Items | Scoring |
|---|---|
| A. Basic Disease Process<br>1. Cancer (Metastatic/Recurrent)<br>2. Advanced COPD<br>3. end-stage liver disease<br>4. kidney dialysis(age ≥65, dialysis for >2 years)<br>5. Advanced cardiac disease<br>6. Neurologic disease with severely reduced function (i.e. stroke, coma, dementia resulting inbed-bound)<br>7. Other life-limiting acute illness (i.e. ARDS ,sepsis, MODS) | Score 2 points EACH |
| B. Concomitant Disease Process<br>1. Cancer (primary)<br>2. Moderate COPD<br>3. Liver cirrhosis<br>4. kidney dialysis (others)<br>5. Moderate congestive heart failure<br>6. Othecondition complicating cure | Score 1 point EACH |
| C. Functional status of patient | Score as specified |

| | |
|---|---|
| Using ECOG Performance Status (Eastern Cooperative Oncology Group)<br>Score Scale<br>Fully active, able to carry on all pre-disease activities without restriction<br>Restricted in physically strenuous activity but ambulatory and able to carry out work of a light or sedentary nature, e.g., light housework, office work.<br>Ambulatory and capable of all self-care but unable to carry out any work<br>activities.<br>2 Capable of only limited self-care; confined to bed or chair<br>3 Completely disabled. Cannot carry on any self-care. | left |
| D. Other criteria to consider in screening<br>The patient:<br>1. Team/patient/family needs help with complex decision-making and determination of goals care<br>2. has unacceptable level of pain<br>3. has uncontrolled psychosocial or spiritual issues<br>4. has frequent visits to the Emergency Department (> 1 x mo for same diagnosis)<br>5. has more than one hospital admission for the same diagnosis in last 30 days<br>6. has prolonged stay in ICU(s) without evidence of progress<br>7. is in an ICU setting with documented poor or futile prognosis<br>8. has prolonged length of stay (>30days) without evidence of progress | Score 1 point EACH |
| Total | |
